# Simultaneous Quantification of Multiple Polycyclic Aromatic Hydrocarbons in Aqueous Media using Micelle Assisted White Light Excitation Fluorescence

**DOI:** 10.1038/s41598-020-65788-2

**Published:** 2020-06-02

**Authors:** John Prakash, Ashok Kumar Mishra

**Affiliations:** 1 Department of Chemistry, Central University of Tamil Nadu, Thiruvarur, 610 005 India; 2 Department of Chemistry, Indian Institute of Technology Madras, Chennai, 600 036 India

**Keywords:** Environmental sciences, Chemistry

## Abstract

Qualitative and quantitative display of multiple fluorescent analytes is made simple and reliable in this micelle assisted methodology. The adopted method involves micelle assisted evincing of *ppb* level of PAHs in water; measurement of total fluorescence (white light excitation fluorescence, WLEF) and data deciphering using multivariate analysis. This protocol yields sensitive and accurate quantification of the cancerous pollutants (PAHs) in aqueous media with Limit of Quantification of the order 1–10 μg/L and accuracy of >98%. The use of WLEF enables the simultaneous acquisition of fluorescence signatures of all the PAHs. It has the additional advantage of being portable, layman-friendly and cost-effective. The optimized amount of surfactants for the simultaneous extraction of PAHs from real samples was estimated as 27.8 mg (19.3 mM) of SDS and 9.1 mg (5 mM) of CTAB. Also, the analytical fidelity of the quantification such as percentage recovery (98 ± 2%), linear dynamic range (2–250 μg/L), RMSEP (<0.5), etc. explains the veracity of methodology.

## Introduction

Polycyclic aromatic hydrocarbons (PAH) are known to be one of the most dreadful and long-lasting pollutants in the living environment^1,2^. PAHs are hydrophobic organic molecules having more than two benzenoid groups in a condensed manner, most of which are carcinogenic to living organisms and hazardous to aquatic life^3,4^. They are one of the main ubiquitous pollutants in the environment, especially in water bodies. The impact of PAHs on the environment and living system is critically reviewed on account of its carcinogenicity and mutagenicity^5^. PAHs reach water bodies from many resources especially from the combustion of fossil fuels, coal tars, incomplete burning of fuels, tobacco products, etc^6^. Once these hydrophobic molecules spread on to the surface water, it may adsorb or condense onto dissolved particulate matter and this dissolved fraction pollutes the water^7^. According to the Environmental Protection Agency (EPA), PAHs need to be carefully monitored in water bodies. Because of low biodegradability, PAH compounds (which may be present in environmental samples in very low amounts) are very tough to eradicate by conventional treatment. The clean-up procedure and quality monitoring are usually required prior determination of PAH contaminants in water. There are many scientific articles that reveal the possibility of dispersive liquid–liquid micro extraction method^8,9^ and solid-phase extraction of PAHs followed by its pre-concentration from water^10^, precipitates^11^ and aerosol^12^. The acceptable limit of PAHs in the environment, especially water bodies, is less than 5 mg L^−1^. Non-polar PAHs occur in water due to the presence of various surfactant media and humic substances and it needs careful attention^13^. LC-MS, GC-MS, HPLC, and spectroscopic techniques have been established for PAHs’ precise quantification in water and soil^14–16^. Among them, fluorescence-based techniques hold more attention because of its simplicity and sensitivity^17,18^.

The mixture of absorbing and fluorescing PAHs in aqueous media make itself complex due to the energy degrading/quenching phenomena such as self-quenching, resonance energy transfer, absorption of exciting photons before reaching the fluorescing zone (inner filter effect), reabsorption of emitted photons, etc^19^. Such complex multiple fluorophoric mixtures (complex multifluorophores) have been analyzed using techniques such as Excitation Emission Matrix Fluorescence (EEMF), Excitation Resolved Synchronous Fluorescence (ERSF), Synchronous Fluorescence Scan (SFS) and Total Synchronous Fluorescence Scan (TSFS)^19,20^. Though these techniques are fairly robust and accurate in predicting analyte composition, they are cumbersome, time-consuming and require scientific expertise for data collection as well as for analysis. Real-time samples often demand *in situ* analysis. For that optical spectrometer should reach the sample, which is almost impossible with conventional spectrometer design. i.e., the desktop design lacks the features of portability and flexibility in optimizing sample-specific geometry. White Light Excitation Fluorescence (WLEF) is evolved recently as a technique to measure total fluorescence in a two-dimensional (2-D) plot. It could investigate multiple fluorophores simultaneously without reconfiguring instrument parameters^21,22^. Also, the WLEF technique requires only a low power light source and hence minimizes the chance of photo-bleaching. These added advantages will help us to integrate a portable device for online/real-time quantification of multiple PAHs in water.

## Objective

In this work, simultaneous real-time analysis of multiple PAHs (multi-probing) in aqueous media is proposed using a Dip Probe Fiber Optic Spectrometer and a highly sensitive WLEF technique with the assistance of the micellar system. The analytical complexity of multifluorophores due to self- quenching, energy transfer, etc. could be nullified under the condition that there is only one fluorophore per micelle on an average. This can be achieved by creating a micelle system using a suitable concentration of surfactant, which in turn restores spectral additive nature. This spectral additive nature of PAHs in the micelle driven system can be used for their simultaneous determination and precise quantification in aqueous media by employing multi-component regression analysis.

## Materials and Methods

### Chemicals

PAHs such as 9-Phenylanthracene (*PA*), Anthracene (*Anth*), 2,3-Benzanthracene (*BA*), Benz[e]acephenanthrylene (*BeA*), Benzo[a]pyrene (*BaP*), 2,3-Benzofluorene (*BF*), Benzo[ghi]perylene (*BP*), Benz[k]fluoranthene (*BkF*), Diphenylanthracene (DPA), Naphtho[2,3a-]pyrene (*NP*) and perylene (*Per*) were procured from Sigma Aldrich and were used without further purification. The PAHs were dissolved in spectral grade acetone (99.5%, Sisco Research Laboratories Pvt. Ltd.) to prepare primary stocks. The solution was further diluted with CTAB (cetyltrimethylammonium bromide, SD Fine-Chemical Ltd) or SDS (sodium dodecyl sulfate, SD Fine-Chemical Ltd) surfactant solutions in water. The surfactant solutions, CTAB and SDS were prepared by dissolving them in triple distilled water (TDW).

### Sample preparation

#### Calibration set of PAHs

The concentrations of PAHs (fluorophores) ranged from 2 to 250 μgL^−1^ in the test solution. The solutions were prepared by transferring the required amount of PAH solution in a volatile solvent (acetone) into a sample container followed by solvent evaporation. It is then dissolved in the required amount of surfactant solution. The concentration of CTAB and SDS in the sample test solution was optimized and maintained as 5 mM and 20 mM respectively for PAHs quantification.

#### Synthetic mixtures for multi-probing

A series of synthetic mixtures (5 components, 3 sets {A, B and C}), were made from the standard stock solutions (1000 ng mL^−1^) for each of the PAHs. The sample matrix is made such that there is enough spectral overlap between their emission profiles. The synthetic mixtures are solubilized randomly using anionic and cationic surfactants as follows, Set A and B are extracted with CTAB (5 mM) and the Set C is with SDS (20 mM). The WLEF intensities of the mixtures are measured using custom-fabricated fiber optic compatible portable spectrometer subsequent to micelle induced disaggregation.

### WLEF instrumentation and data acquisition

In our previous publication, WLEF has been introduced as the 2-D analogue of the 3-D total fluorescence spectrum, EEMF, which collects total fluorescence response from the sample and display a plot of emission intensity against wavelength^21^. WLEF intensity at a specific wavelength is proportional to the excitation efficiency, integral product of absorption profile of molecule and the lamp profile of an exciting source.

WLEF has two main advantages, viz.,

- It features simultaneous excitation (WLE) of all the fluorophores
- WLE improves emission intensity by populating more molecules in the excited state.

$$\begin{array}{c}\begin{array}{ccc}{I}_{WELF} & \propto  & {\int }_{WLE}{A}_{\lambda }{L}_{\lambda }d\lambda \\ {I}_{WELF} & \propto  & {\int }_{WLE}(\mathop{\sum }\limits_{i=1}^{n}{A}_{\lambda ,i}{L}_{\lambda })d\lambda \end{array}\end{array}$$

The reduction of data dimension (3D to 2D), fast data acquisition, spectral veracity, sim Dip Probe Fiber Optic spectrometerplicity in analysis, cost-effectiveness and portability are additional features of the WLEF method. The perks of WLEF are already exploited in monitoring quality of fuel blends^23^, biofluids^24^, etc.

A fiber optic compatible and portable Dip Probe Fiber Optic Spectrometer^25^ was fabricated for the WLEF measurement using a white light excitation source (270–900 nm, W-X lamp); sensitive CCD-Diode Array Detector (DAD, QE9000-Pro) and UV–VIS XRS Solarization Resistant fibers (1 m, transmission range:180–900 nm, diameter: 600 μm and NA: 0.22). The components were purchased from Ocean Optics Inc. The WLEF spectra were collected using a personal computer connected to a fiber-optic spectrometer. The Ocean View software procured from Ocean Optics Inc. eases data acquisition.

**Figure 1:**
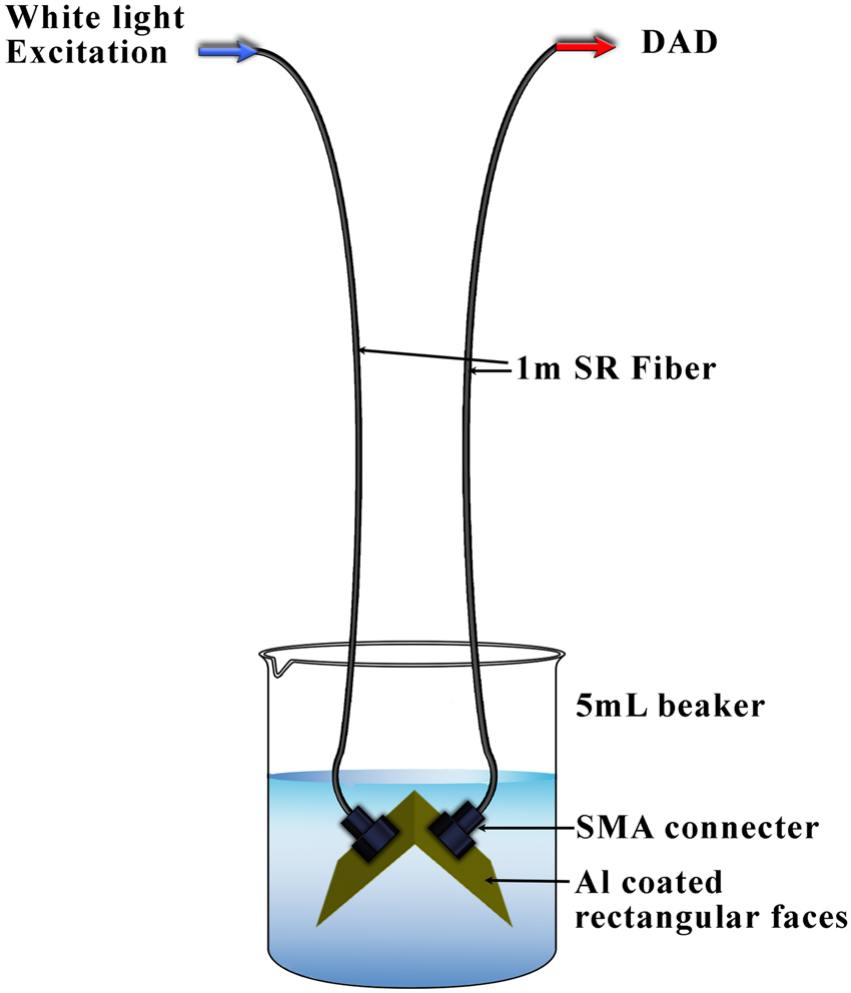
The optical design of Dip Probe Fiber Optic Spectrometer for WLEF measurement.

The schematic optical scheme of the Dip Probe Fiber Optic Spectrometer is shown in Fig. 1 (A photograph of the Dip Probe design is given as ESI-1). The dip probe is fabricated such that the fluorescence emission was collected at the right-angled geometry to the excitation beam, which minimizes spectral contamination by reflected and transmitted light. The volume of the test solution required for the measurement is 5 mL and the path length maintained as 10 mm. Optimized instrument parameters for WLEF measurement are 10 seconds of spectral integration, averaged over 5 spectra and boxcar smoothening across 5 consecutive wavelengths. These optimized parameters gave reasonable highest WLEF intensity for each species, which was chosen for the entire analysis.

### Multivariate analysis of WLEF data

The WLEF intensity at each wavelength depends on two variables, viz., concentrations of species and their respective WLEF intensities, i.e., the data matrix has a bilinear structure. The uniqueness of Multivariate Curve Resolution–Alternating Least Square (MCR-ALS) analysis is that it provides analyte-specific pure spectral (instrument) responses along with their abundance as concentration profiles in the output. Though the exploratory methods like independent component analysis (ICA), principal component analysis (PCA), etc. are good for resolving bilinear data, the MCR-ALS algorithm was preferred over them to analyze the WELF data matrix. Also, ICA or PCA methods lack a genuine chemical or scientific model. MCR-ALS algorithm alternatively optimizes both the spectral and concentration profiles in each iterative cycle. Iterative methods do not require a structured concentration direction. MCR always furnishes a simple bilinear description based on the variation in the data especially the natural properties of the problem of interest. MCR-ALS algorithm has given the freedom of the incorporating chemical and mathematical constraints to make the data analysis task efficient. These characteristics make MCR outputs legible to non-chemometricians^26–28^.

The PLS-Toolbox 5.0.3 written in MATLAB language was used for MCR-ALS analyses as follows. The WLEF intensity of the mixture of PAHs at a particular emission wavelength, ***W***_***i***_ is given by

$${{\boldsymbol{W}}}_{{\boldsymbol{i}}}=\mathop{\sum }\limits_{{\boldsymbol{i}}=1}^{{\boldsymbol{n}}}{{\boldsymbol{c}}}_{{\boldsymbol{i}}}{{\boldsymbol{w}}}_{{\boldsymbol{i}}}$$

where ***w***_***i***_ and ***c***_***i***_ are respectively the WLEF intensity and the fractional concentration of the ***i***^th^ component. The value of *‘****i****’* is ranging between 1 and n, the total number of the component present in the mixture. Hence the quantification needs to estimate concentration ***c***_***i***_’s along with the unique molecular feature ***w***_***i***_. A set of ‘n’ linear equations with ‘n’ fractional coefficients can be resolved as follows.

The relationship between the WLEF data matrix ***W***_***i***_ is bilinear in nature, i.e., it is the sum of the product of the concentration of each PAHs and the respective spectra as expected.

$${{\rm{W}}}_{{\rm{i}}}{\rm{=}}{\rm{C}}{{\rm{S}}}^{{\rm{T}}}{\rm{+}}{\rm{E}}$$

where ***W*** is the WLEF spectra of ***I*** samples against ***J*** wavelengths; ***C*** is the concentration of ***K*** number of PAHs in the analytes; ***S***^***T***^ are the pure WLEF spectra, its rows contain the pure spectra of respective species; ***E*** is the calibration error^26^.

Prior to MCR analysis, the number of components and the spectral window are iteratively optimized by means of PCA followed Evolving Factor Analysis (EFA) procedures^29^. The initial estimation for the spectral profile (determination of the purest variables) is done using EFA followed by MCR in order to initiate the iterative ALS procedure. The initial estimation for the spectrum gives an unconstrained least square solution for the concentration.

$${\boldsymbol{C}}={\boldsymbol{D}}{({{\boldsymbol{S}}}^{{\boldsymbol{T}}})}^{+}$$

where, (***S***^***T***^)^+^ = ***S***(***S***^***T***^ ***S***)^−1^, pseudo-inverse of ***S***^***T***^

Similar estimations can be done on concentration in the ALS cycles; such iteration yields new matrices of ***C*** and ***S***^***T***^.

## Results and Discussion

### Selection of PAHs and its sensitivity in WLEF measurement

The PAHs were chosen based on their insoluble nature and cancerous properties (Table 1). Since they last in the environment for quite a long time (half-life ~ 6 yrs), these living carcinogens are monitored in aqueous media. The acceptable limit of PAHs in the environment, especially water bodies, is less than 1 to 5 mg L^−1^.

**Table 1:** The solubility of PAHs in water and their associated health issues^34–38^.

PAH	Solubility in water (μg/L)	Health issue
2,3-Benzanthracene (*BA*, Naphthacene)	1.51	Dermal irritant and very toxic to aquatic life
2,3-Benzofluorene (*BF*)	2.38	Endocrine disruptors
9-Phenylanthracene (*PA*)	Insoluble	Eye irritant and toxic to aquatic life
Anthracene (*Anth*)	0.044	Lung cancer, coughing and wheezing
Benz[e]acephenanthrylene (*BeA*)	1.49	Carcinogen and very toxic to aquatic life
Benz[k]fluoranthene (*BkF*)	0.801	Carcinogen and irritant
Benzo[a]pyrene (*BaP*)	1.62	Carcinogen and causes respiratory tract irritation
Benzo[ghi]perylene (*BP*)	2.61	Very toxic to aquatic life
Naphtho[2,3a-] pyrene (*NP*)	Insoluble	Dermal and oral toxicity
Perylene (*Per*)	0.41	Affects cardiovascular system

**Figure 2:**
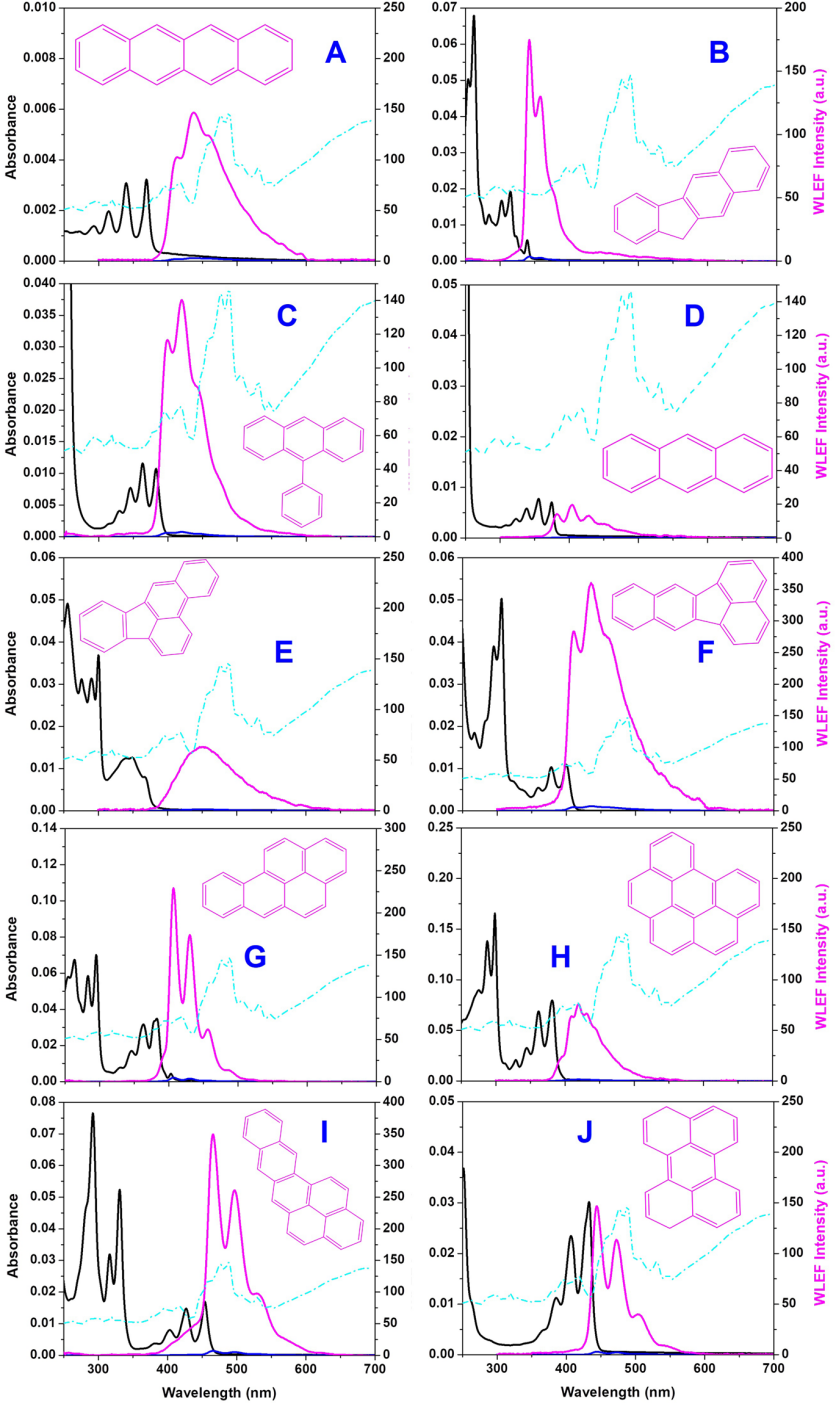
UV-VIS electronic absorption spectra (1 μM, black) of PAHs, lamp profile (cyan) for electronic white excitation and WLEF spectra of PAHs (10 nM) in aqueous media in presence (magenta) and absence (blue) 5 mM CTAB surfactant media {[**A**] 2,3-Benzanthracene (BA), [**B**] 2,3-Benzofluorene (BF), [**C**] 9-Phenylanthracene (PA), [**D]** Anthracene (Anth), [**E]** Benz[e]acephenanthrylene (BeA), [**F**] Benz[k]fluoranthene (BkF), [**G**] Benzo[a]pyrene (BaP), [**H**] Benzo[ghi]perylene (BP), [**I**] Naphtho[2,3a-]pyrene (NP) and [**J**] Perylene (Per)}.

The WLEF spectra of selected PAHs measured in 5 mM CTAB are shown in Fig. 2.

The merits of this analytical procedure for each PAH are independently established by making a linear plot of WLEF intensity against the amount of PAH. WLEF intensity in the concentration ranges from 10 to 100 nM. The WLEF spectra and their respective calibration plots of selected PAHs are presented as ESI-2. The Linear Quantification Range (LQR), Pearson’s regression coefficient (R^2^) and Limit of Detection (LOD) were determined for each PAH and gathered in ESI-3.

The LDR for the analysis of PAHs by WLEF ranged from 2.5 to 250 μgL^−1^ with very good correlation (R^2^ ~0.9989 to 0.9999). The LOD of various PAHs was found to be between 0.1 and 2 μgL^−1^. Figure 2 clearly reveals that there is a robust spectral overlap among the emission spectrum of PAHs. The linearity plot of WLEF intensity against concentration in the analytical range (ESI-2) verifies that the possibility of energy transfers, inner filter effects, etc., is negligible in micellar media. In this regard, though the simultaneous quantification of multiple components has a good impact, a suitable data collection methodology followed by logical analysis is also required.

### Micelle assisted probing of multiple PAHs in aqueous media

The micelle assisted multi-probing aims at instantaneous quantitative visualization of multiple PAHs in real samples. It involves 3 steps, viz., (a) micellar dissolution of PAHs to enhance the fluorescence as well as remove spectral interferences, (b) measurement of WLEF to obtain total fluorescence features in a very short time and (c) spectral deconvolution using chemometrics to provide qualitative and quantitative information.

#### *Micelle assisted dissolution of PAHs*

Micelles have two roles in this method, (a) minimize the problems of non-linearity and regain spectral additivity and (b) provide a hydrophobic environment to the analyte and make it visible. Micelle scavenges each PAH from aqueous media to its micellar cavity and increases the average distance between them which leads to enhanced sensitivity, reduced non-radiative energy transfer, and negligible collisional quenching^3^*.*

**Figure 3:**
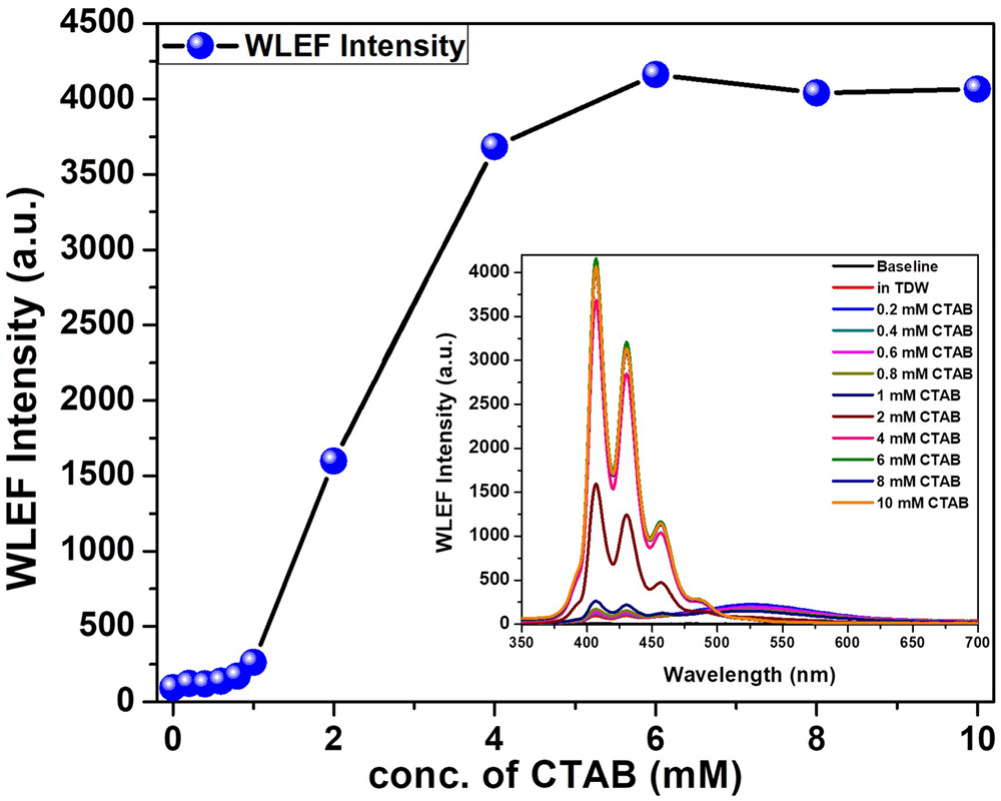
The variation of WLEF intensity at its emission maxima of benzo[a]pyrene (*BaP*, 0.75 µM) with surfactant (CTAB; 1 to 10 mM) concentration measured in a custom designed Dip Probe Fiber Optic Spectrometer (spectral intensity is averaged for 3 measurements). Inset figure shows the WLEF spectra for the same.

Micellar solubilization of PAHs (*BaP* as a representative example, which is insoluble in water) was studied by fixing the concentration of it and varying the amount of surfactant, CTAB (cmc = 0.96 mM; CAN = 62), in the test solution (Fig. 3). Enhanced solubility or fluorescence is observed for the sample above cmc of surfactant, due to the micellar scavenging of fluorescent and hydrophobic PAH. Saturation in fluorescence intensity was observed above 5 mM of CTAB (micelle concentration, [M] =65 μM). So the optimum concentration of the micellar medium, CTAB, was selected as 5 mM to scavenge PAH from aqueous media with minimal intensity quenching effects. Similar study has been done with an anionic surfactant, SDS (cmc = 8.3 mM; CAN = 63) and optimized concentration was found to be 19.3 mM ([M] =175 μM; ESI-4a & b).

$$P(n)=\frac{{x}^{n}{e}^{-x}}{n!}$$

The Poisson’s distribution was used to predict the number probability, P (n), of PAH molecules present per micelles^30,31^.

$$P(n)=\frac{{x}^{n}{e}^{-x}}{n!}$$

The variables ‘x’ and ‘n’ are the PAH to micelle ratio and the number of PAH respectively. For the given micellar (CTAB, 5 mM) and PAH (0.75 μM) concentrations, the chance of one PAH being inside a micelle is about 1%. Likewise, the probability of two PAHs in a single micelle is 4.36 × 10^−5^. The table, ESI-5, clearly indicates that the probability of finding two or more PAHs in the same micelle is rarest or can be omitted.

The fluorescence negating phenomena of resonance energy transfer and the dynamic collisional quenching *etc*. are thus nullified by pushing each PAHs into separate micelles and hence by increasing the average spacing between any two PAHs. In addition, micellization enhances solubilization of hydrophobic PAHs and hence fluorescence intensity. The effect of the second fluorophore on the calibration curve of a target fluorophore was established by gradually varying the concentration of second (interfering) fluorophore. The interference between different fluorophore and spectral additivity of PAH mixtures were also investigated and were found to be negligible in micellar media. The optimized concentration of the surfactant regains the spectral additivity of target as well as interfering fluorophores with an analytically relevant range (2 to 250 μgL^−1^). The amount of surfactants required for the complete evincing of multifluorophoric mixture (sample volume - 5 mL) was 9.1 mg (5 mM) and 27.8 mg (19.3 mM) respectively for CTAB and SDS. Since the pH and presence of salts (ionic strength) can significantly alter the cmc, the optimized surfactant concentration may vary as a function of the concentration of other ionic species in the real system. This must be factored in while taking real-life samples. Non-ionic surfactants with a zeta potential of the micelles close to zero may be the most ideal choice.

#### *WLEF spectral data acquisition and chemometric analysis*

Aqueous analyte containing a mixture of PAHs of interest, [5 - component synthetic mixture(s)] is solubilized in a micellar media. It is then examined under the white light excitation; each component emits a unique fluorescence spectrum. Since the micelle induced solubilization eliminates the fluorescence degrading possibilities such as energy transfer, inner filter effects, etc.; hence, the WLEF spectra of the multiple PAHs regain the spectral additivity feature. A multivariate analysis based spectral deciphering of these mixtures into individual emission can be used for the identification of individual components and their quantification.

MCR–ALS analyses can decompose the WLEF spectral data matrix into concentration and spectral data. To get meaningful information a series of logical and mathematical constraints are employed. Initially, non-negativity constraint is applied to both spectral and concentration profiles because of their real nature. Since the spectra of PAHs are having vibrational structures, unimodality constraints cannot be a logical choice, so unimodality constraint is applied only to the concentration profile^29,32^. The correlation constraint is smartly introduced to predict the exact concentration of analytes in an unknown environment.

**Figure 4:**
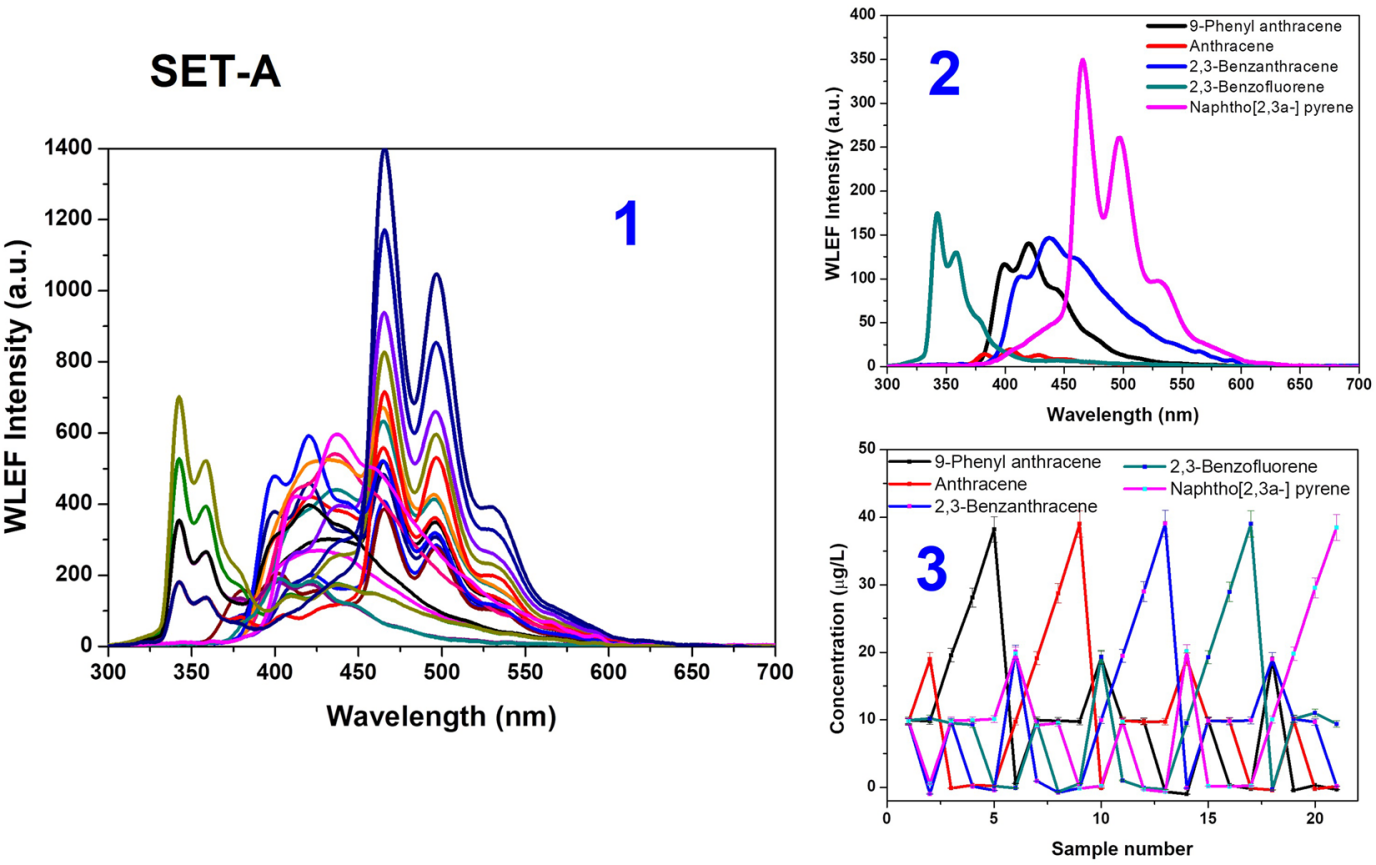
Spectral deciphering and quantification of complex mixtures of analyte (Set A) using MCR-ALS multivariate analysis. (1) WLEF spectra of analyte, (2) spectral information of analytes and (3) their concentration profiles with 3% error bar [WLEF spectra are averaged for 5 measurements].

A soft MCR-ALS procedure performed on calibration data with non-negativity constraints on both concentrations and spectra profiles, and unimodality on concentration profile. PCA initially estimates the number of components as 5 which captures the maximum variation of about 99.9% for all the three sets. This is matching with the composition of the spiked samples as well. EFA yields the spectral window (variable) in which the analytes are present; only two components have less overlap with the rest of the spectra, the other three are completely overlapped. The MCR-ALS resolved 5 species for all the three sample sets (A, B and C). Spectral deciphering and quantification of multiple analytes in complex mixture, Set A (mixtures Set B and C are shown in ESI-6a & b) using MCR-ALS multivariate analysis is shown in Fig. 4. The exactitude of resolution is evaluated by considering 3 parameters, viz., Standard Deviance of residuals, the fitting error and the variance and is tabulated in ESI-7.

The correlation constraint creates an MCR-ALS calibration model, which can be adopted for the quantitative visualization of the PAHs in real samples; even unknown spectral interferences have existed. Prior knowledge of the spectral nature helps us to identify the PAH along with its amount in the mixture accurately. Percentage (%) recovery of each PAH present in the 3 sets of calibration samples was determined (ESI-8) using the following relations.

$$relative\,{\rm{ \% }}\,error=\left(\frac{x-x{}^{{\prime} }}{x}\right)\times 100$$

$$ \% \,Recovery=(100-relative\, \% \,error)$$

where ***x*** is the spiked amount and ***x’*** is the recovered amount of PAH.

The root mean square error in prediction (RMSEP) of the analyte combinations for each sample determined using the following relation.

$$RMSEP=\sqrt{\frac{{\sum }_{i=1}^{n}{(x-{x}_{i})}^{2}}{n}}$$

Relative percentage recoveries of sample mixtures were found to be 98 ± 2% and lack-of-fit in all the analytes were well below 1%. So, this micelle assisted methodology for probing multiple PAHs (*multi-probing*) in aqueous media using WLEF could be a low cost, portable option. Also, this methodology can be easily adapted for the *real-time* monitoring of PAHs in different water bodies.

### Real sample analysis (% recovery of PAHs)

**Figure 5:**
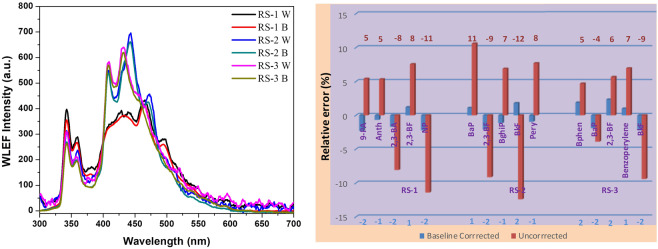
(**a**) WLEF spectra of 3 mixtures of PAHs spiked river samples (RS) without (W) and with baseline correction (B) measured in real time using SDS micellar media. (**b**) Relative prediction error in the percentage recovery of spiked mixtures in three RS. The red histogram (with % error) shows uncorrected spectral deconvolution using MCR-ALS and blue histograms for the respective baseline corrected [spectral measurements are triplicated and deviation from the mean is less than 2%].

The real analytical system carries complexity with respect to matrix fluorescence (mainly, humic substance) and as well as multiple fluorophoric components. The reliability of the methodology was tested by a river water sample obtained from *Vettar-*river spiked with 3 synthetic mixtures of PAHs. The amount of SDS used to extract the total fluorescence was 30 mg (optimized value 27.8 mg). SDS is adopted for real sample analysis because of its environment-friendly nature. The WLEF spectra of the spiked samples are resolved using the developed model (Fig. 5). The river water sample was collected and filtered through disposable syringe filters (MILLEX, 33 mm, and 0.22 μm) to remove particulate matters, followed by PAHs spiking in order to minimize the scattering and particulate matters. The humic substance present in the fairly clear river water sample was found to be 142 ± 5 ppm^33^. Correlation constraint eases the adoption of developed MCR-ALS model to predict the quality of water even in the presence of background fluorescence interference from humic substances and other fluorescing contaminants. The calibration model designed for a test sample has been used to predict the quantity of the ‘unknown’ mixture in river water samples.

The percentage recovery of each PAHs present in the 3 sets of samples are tabulated with its figures of merit (Table 2). The RMSEP values show that the spectral subtraction will predict the sample composition with more precision. The spectral subtraction using a suitable baseline (TDW is used here) removes unwanted and inherent scattering components such as Rayleigh and Raman scatterings. The percentage of recovery studies show that baseline-corrected measurement has a feeble deviation from the original values (~2%) and the RMSEP is well below 0.3 units. These parameters explain the reliability and fidelity of the methodology in real samples. The uniqueness of spectral features and the availability of fluorescence spectral libraries of PAHs widen the scope of our methodology. The simplicity of instrumentation, data collection in no time, ease of data processing, portability of equipment, etc. are the additional features of this micelle assisted *multi-probing*. It allows *real-time* monitoring and simultaneous quantification of a mixture of PAHs in water bodies. This proof-of-concept work well with low scattering samples (even PAH spiked river samples) and merely pure with respect to other ionic contaminants and low salinity samples.

**Table 2:** Root mean square error in prediction (RMSEP) of the analyte composition in the river water samples (RS).

PAHs	Spiked (nM)	Estimated	Deviation	Square	Estimated	Deviation	Square
		Raw Sample (W)			Baseline Corrected (B)		
	River Water Sample-1 (RS-1)						
9-phenylanthracene	10	10.23	−0.23	0.052_9_	9.46	0.54	0.291_6_
Anthracene	25	25.17	−0.17	0.028_9_	23.67	1.33	1.768_9_
2,3-Benzanthracene	15	15.26	−0.26	0.067_6_	16.21	−1.21	1.464_1_
2,3-Benzofluorene	20	19.76	0.24	0.057_6_	18.49	1.51	2.280_1_
Naphtho[2,3a-]pyrene	5	5.11	−0.11	0.012_1_	5.57	−0.57	0.324_9_
**RMSEP**				**0.209**_**3**_			**1.107**_**2**_
	**River Water Sample-2 (RS-2)**						
Benzo[a]pyrene	10	9.89	0.11	0.012_1_	8.94	1.06	1.123_6_
2,3-Benzofluorene	15	15.31	−0.31	0.096_1_	16.37	−1.37	1.876_9_
Benzo[ghi]perylene	25	25.29	−0.29	0.084_1_	23.28	1.72	2.958_4_
Benz[k]fluoranthene	5	4.91	0.09	0.008_1_	5.62	−0.62	0.384_4_
Perylene	20	20.19	−0.19	0.036_1_	18.46	1.54	2.371_6_
**RMSEP**				**0.217**_**5**_			**1.320**_**2**_
	**River Water Sample-3 (RS-3)**						
Benz[e]acephenanthrylene	25	24.65	0.35	0.120_4_	23.83	1.17	1.368_9_
Benzo[a]pyrene	10	10.19	−0.19	0.036_1_	10.39	−0.39	0.152_1_
2,3-Benzofluorene	15	14.75	0.25	0.062_5_	14.15	0.85	0.722_5_
Benzo[ghi]perylene	20	19.8	0.20	0.040_0_	18.61	1.39	1.932_1_
Benz[k]fluoranthene	5	5.11	−0.1	0.012_1_	5.47	−0.47	0.220_9_
**RMSEP**				**0.232**_**9**_			**0.937**_**7**_

## Conclusions

The micelle assisted fluorescence enhancement followed by multivariate deciphering methodology may be devised for the real-time analysis of complex, but transparent analytes. The micellar scavenging made the ‘invisible’ carcinogens (PAHs) visible by providing a more hydrophobic environment and increasing solubility of PAHs. The optimized amount of surfactants required for the micellar extraction of PAHs is determined as 19.3 mM for SDS and 5 mM for CTAB respectively. WLEF of micellar extracted multifluorophoric systems regains spectral addition feature by optimizing the maximum number of PAHs in a micelle to unity and hence minimize self-quenching, energy transfer, etc. The WLEF spectra of mixtures are deciphered into quality and quantity information using multivariate MCR-ALS. Also, the LOD of various PAHs in water was found to be of the order of micrograms per liter (*sub*nanomolar). The reliability and fidelity of the methodology are tested with real samples which yield more than 97% of accuracy with ~0.3 of RMSEP. The issues of Rayleigh and Raman scattering in the data set are easily removed in this methodology by baseline subtraction. In summary, Micelle assisted extraction followed by MCR–ALS analysis of bilinear structured WLEF data can easily probe multiple fluorescent components in a facile manner. Also its application potential under various matrix conditions like scattering/intrinsic fluorescence/pH/salinity/hardness etc. are yet to be explored.

## Supplementary information

Supplementary information.
