# Supplementary material for: Simultaneous Quantification of Multiple Polycyclic Aromatic Hydrocarbons in Aqueous Media using Micelle Assisted White Light Excitation Fluorescence
Source: Sci Rep. 2020 Jun 2;10:8921. doi: 10.1038/s41598-020-65788-2 (PMC7265557; doi:10.1038/s41598-020-65788-2)
Supplement: Supplementary file 1 — Supplementary information. [file 41598_2020_65788_MOESM1_ESM.docx]

**Simultaneous Quantification of Multiple Polycyclic Aromatic Hydrocarbons in Aqueous Media using Micelle Assisted White Light Excitation Fluorescence**

^1^John Prakash and ^2^Ashok Kumar Mishra*

First author

John Prakash

Assistant Professor

Department of Chemistry,

Central University of Tamil Nadu,

Thiruvarur 610 005

India

[johnprakash@cutn.ac.in](mailto:johnprakash@cutn.ac.in)

Second Authorand **Corresponding author**

Ashok Kumar Mishra

Professor

Department of Chemistry

Indian Institute of Technology Madras,

Chennai-600 036

India

[mishra@iitm.ac.in](mailto:mishra@iitm.ac.in)

Electronic Supplementary Information

**ESI-1**: Photograph of one of the main components of the dip-probe spectrometer, a custom-designed sample holder that can be dipped into the sample solution (minimum volume required is 5 mL) and measure total fluorescence.


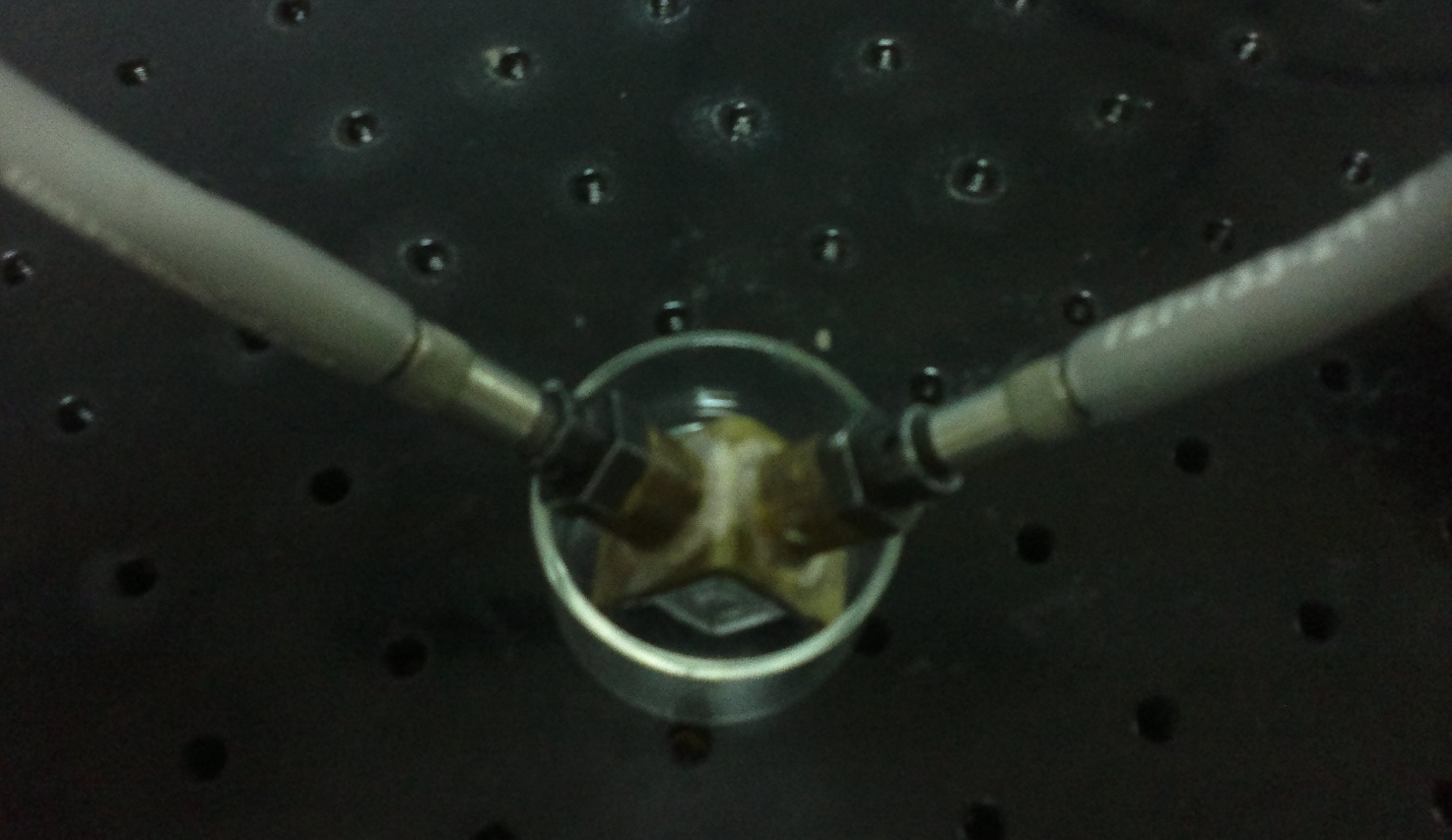


**ESI-2**: WLEF spectra (a) and their respective calibration plots (b) of WLEF intensity maximum against concentration of the chosen PAHs

1. 2,3- Benzanthracene

1. 2,3-Benzofluorene

1. 9-Phenylanthracene

1. Anthracene

1. Benz[e]acephenanthrylene

1. Benz[k]fluoranthene

1. Benzo[a]pyrene

1. Benzo[ghi]perylene

1. Naphtho[2,3a-] pyrene

1. Perylene

**ESI-3**: Analytical figures of merit of single fluorophore in micelle assisted WLEF

| PAHs | Emission Max (nm) | LOD (μg/L) | LQR (μg/L) | R^2^ |
| --- | --- | --- | --- | --- |
| 2,3-Benzanthracene (*BA*) | 437 | 0.13 | 2.3 to 228 | 0.999_8_ |
| 2,3-Benzofluorene (*BF*) | 342 | 0.12 | 2.2 to 216 | 0.999_9_ |
| 9-Phenylanthracene (*PA*) | 420 | 0.14 | 2.5 to 254 | 0.999_9_ |
| Anthracene (*Anth*) | 404 | 0.13 | 1.8 to 178 | 0.999_0_ |
| Benz[e]acephenanthrylene (*BeA*) | 450 | 1.61 | 2.5 to 252 | 0.999_1_ |
| Benz[k]fluoranthene (*BkF*) | 436 | 0.10 | 2.5 to 252 | 0.999_9_ |
| Benzo[a]pyrene (*BaP*) | 407 | 0.31 | 2.5 to 252 | 0.999_1_ |
| Benzo[ghi]perylene (*BP*) | 419 | 0.42 | 2.8 to 276 | 0.999_4_ |
| Naphtho[2,3a-]pyrene (*NP*) | 466 | 0.07 | 3.0 to 302 | 0.999_9_ |
| Perylene (*Per*) | 472 | 0.79 | 2.5 to 252 | 0.999_9_ |

**ESI-4a**: WLEF spectra of Diphenylanthracence (0.75 µM) in Sodium Dodecyl sulphate surfactant (SDS; 1 to 40 mM) recorded in a custom designed dip probe fiber optic fluorimeter (Spectral intensity is averaged for 3 measurements).

**ESI-4b**: The variation of WLEF intensity of Diphenylanthracene (λ_max_=426 nm, 0.75 µM) with SDS (1 to 40 mM) concentration measured in a custom designed dip probe fiber optic fluorimeter (Spectral intensity is averaged for 3 measurements).

**ESI-5**: Probability, P(n), of finding number ‘n’ of molecules in a micelle

| **N** | **P(n)_CTAB** | **P(n)_SDS** |
| --- | --- | --- |
| 1 | 9.39 X 10^-3^ | 2.45 X 10^-3^ |
| 2 | 4.36 X 10^-5^ | 3.00 X 10^-6^ |
| 3 | 1.35 X 10^-7^ | 2.45 X 10^-9^ |
| 4 | 3.15 X 10^-10^ | 1.50 X 10^-12^ |
| 5 | 5.85 X 10^-13^ | 7.35 X 10^-16^ |

**ESI-6a**: Spectral deciphering and quantification of complex mixtures of analyte (Set B) using MCR-ALS multivariate analysis. (1) WLEF spectra analyte (21 combinations), (2) spectral information of analytes and (3) their concentration profiles [WLEF spectra is averaged for 5 measurements].


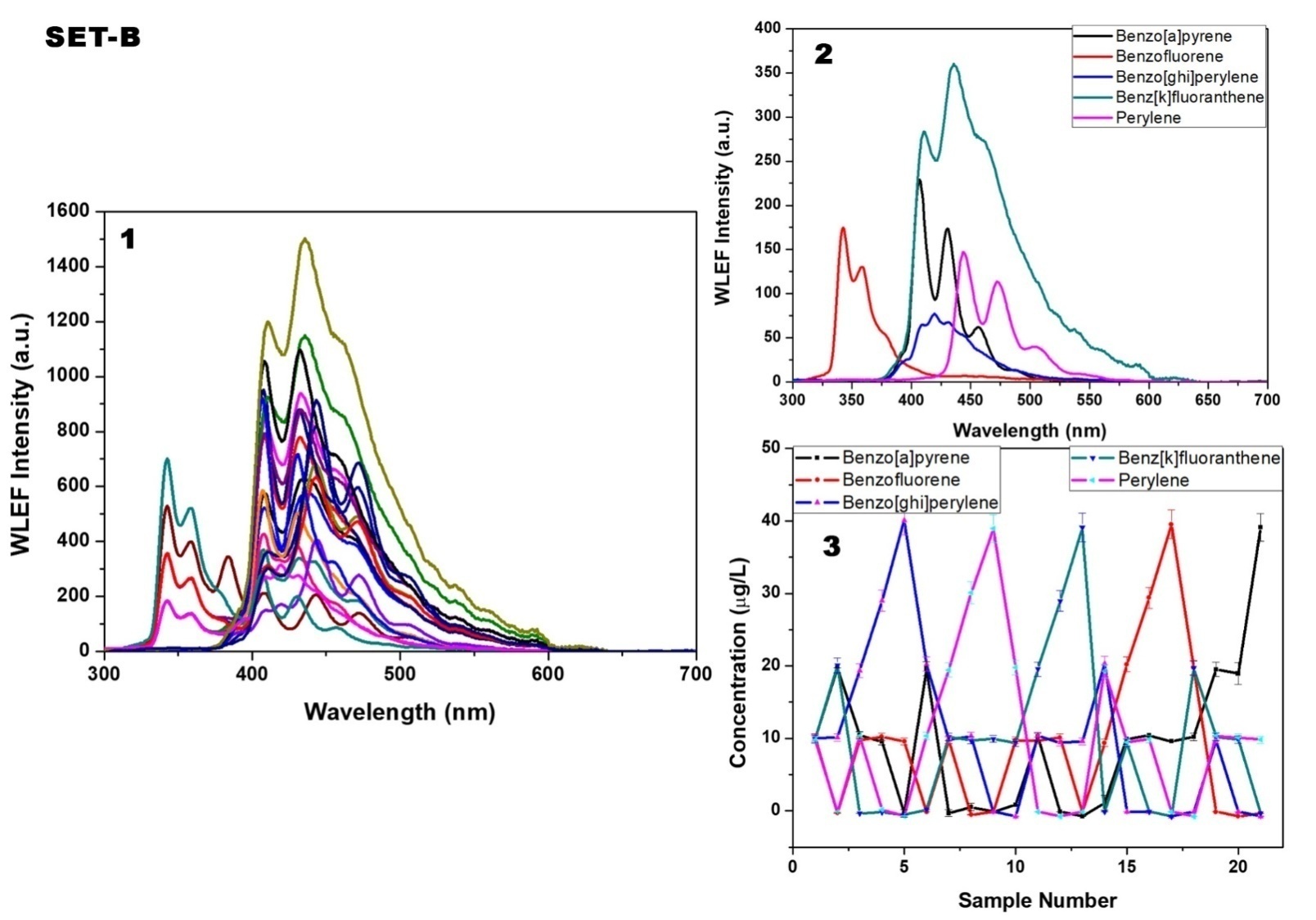


**ESI-6b**: Spectral deciphering and quantification of complex mixtures of analyte (Set C) using MCR-ALS multivariate analysis. (1) WLEF spectra analyte (21 combinations), (2) spectral information of analytes and (3) their concentration profiles [WLEF spectra is averaged for 5 measurements].


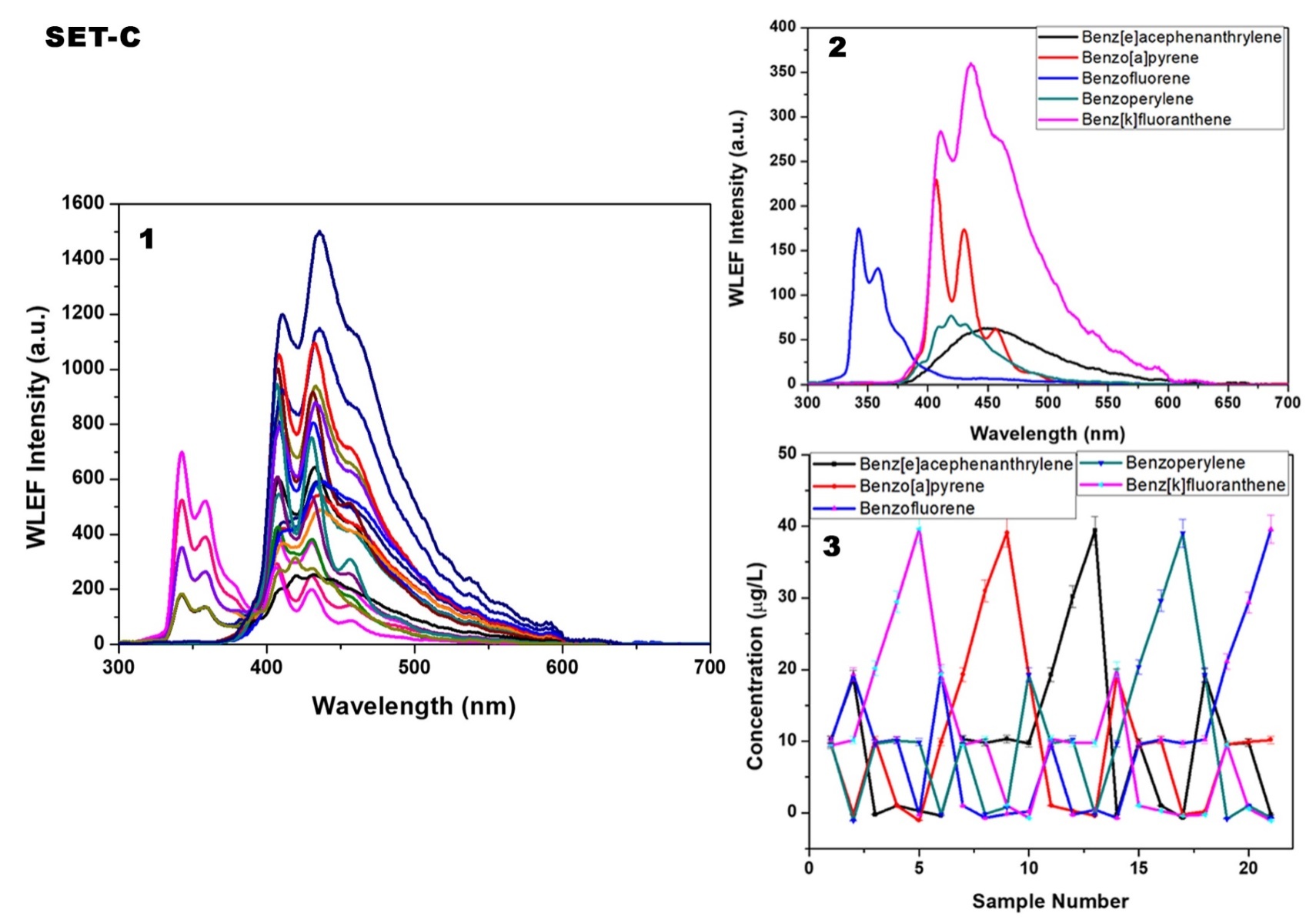


**ESI-7**: Evaluation of precision in resolving WLEF data matrix into spectral and concentration profiles

|  | Standard deviance | Explained variance  (%) | Fitting error  (%) |
| --- | --- | --- | --- |
| Set A | 0.0135 | 99.934 | 0.068 |
| Set B | 0.0042 | 99.998 | 0019 |
| Set C | 0.0078 | 99.962 | 0.036 |

**ESI-8** The analytical figures of merits (a) relative percentage of recovery for each PAHs in each analytes and (b) root mean square deviation (RMSEP) for each analytes.

^$^ relative percentage of recovery for each PAHs is averaged for 13 combinations in 21 analytes

^#^ root mean square deviation (RMSEP) for each samples are averaged for 21 analytes

| Analyte | PAHs | Relative % Recovery^$^ | RMSEP^#^ |
| --- | --- | --- | --- |
| Set A | 9-phenyl Anthracene | 97.39 | 0.73305 |
|  | Anthracene | 96.96 |  |
|  | 2,3-Benzanthracene | 97.80 |  |
|  | 2,3-Benzofluorene | 96.23 |  |
|  | Naphtho[2,3a-]pyrene | 97.75 |  |
| Set B | Benzo[a]pyrene | 99.97 | 0.43418 |
|  | Benzofluorene | 97.63 |  |
|  | Benzo[ghi]perylene | 97.48 |  |
|  | Benz[k]fluoranthene | 97.54 |  |
|  | Perylene | 97.61 |  |
| Set C | Benz [e]acephenanthrene | 97.29 | 0.5393 |
|  | Benzo[a]pyrene | 97.09 |  |
|  | Benzofluorene | 97.25 |  |
|  | Benzoperylene | 97.53 |  |
|  | Benz[k]fluoranthene | 97.38 |  |
